# Effects of *Melissa officinalis* (lemon balm) consumption on serum lipid profile: a meta-analysis of randomized controlled trials

**DOI:** 10.1186/s12906-024-04442-0

**Published:** 2024-04-04

**Authors:** Kasra Shahsavari, Mohammad Reza Shams Ardekani, Mahnaz Khanavi, Tannaz Jamialahmadi, Mehrdad Iranshahi, Maede Hasanpour

**Affiliations:** 1 School of Medicine, Tehran University of Medical Sciences, Tehran, Iran; 2 Department of Pharmacognosy, Faculty of Pharmacy, and Persian Medicine and Pharmacy Research Center, Tehran University of Medical Sciences, Tehran, Iran; 3 Biotechnology Research Center, Pharmaceutical Technology Institute, Mashhad University of Medical Sciences, Mashhad, Iran; 4 Department of Nutrition, Faculty of Medicine, Mashhad University of Medical Sciences, Mashhad, Iran

**Keywords:** *Melissa officinalis*, Lemon balm, Dyslipidemia, Lipid profile, Clinical trials, Meta-analysis

## Abstract

**Background:**

According to traditional medicine, *Melissa officinalis* L., (lemon balm) has been known to remove harmful substances from the blood and is considered a cardiac tonic. Therefore, its use as a cardiovascular remedy may explain the lipid-lowering effects of lemon balm. Dyslipidemia can be considered as a significant preventable risk factor for atherosclerosis, coronary heart disease and type 2 diabetes. The present study is the first meta-analysis to investigate the effects of *M. officinalis* administration on serum levels of high-density lipoprotein cholesterol (HDL), low-density lipoprotein cholesterol (LDL), triglyceride (TG) and total cholesterol (TC).

**Methods:**

From inception to October 2023, a thorough search through literature was conducted using PubMed, Scopus, and Web of Science. The inclusion criteria of this study were randomized controlled trials, with or without blinding which provided adequate data for each group at the beginning and end of the follow-up period. Meta-analysis was performed on randomized controlled trials using Comprehensive Meta-Analysis (CMA) V4 software. Risk of bias in the selected studies was examined according to the revised Cochrane risk-of-bias tool for randomized trials. Begg’s funnel plot symmetry status, Begg’s rank correlation, and Egger’s weighted regression tests were employed to evaluate potential publication bias.

**Results:**

The meta-analysis comprised of 5 randomized controlled trials with a total of 302 patients. The findings of the meta-analysis indicated that the consumption of lemon balm had a significant decrease in TG (SMD (95% CI): -0.396(-0.620, -0.173), *p-*value *=* 0.001), TC (SMD (95% CI): -0.416 (-0.641, -0.192), *p*-value < 0.001) and LDL (SMD (95% CI): -0.23(-0.45, -0.008), *p* < 0.05) levels compared to the placebo group. While it had no statistically significant effect on HDL level (SMD (95% CI): 0.336(-0.091, 0.767), *p*-value = 0.123). No significant and detectable publication bias was found in the meta-analysis. Additionally, all included clinical studies demonstrated a low risk of bias for missing outcome data and selection of the reported results. The robustness of the results was demonstrated by a sensitivity analysis using the one-study remove method.

**Conclusions:**

The findings of this meta-analysis provide evidence that lemon balm may be administered as a safe and beneficial herbal medicine for reducing TC, TG and LDL levels. According to the pooled results of 5 studies with a total of 302 patients, lemon balm intake had no significant effect on HDL level. This study reinforces the notion that lemon balm may have a substantial impact on serum lipid profile as a potential remedy in cases of dyslipidemia. The main concern of our research is the limited number of eligible studies and the relatively small population size of each individual study. The patients of these studies had different types of diseases and metabolic syndromes. However, the meta-analysis was sufficiently powered to detect the considerable effects of lemon balm in the combined population regardless of type of diseases.

**Supplementary Information:**

The online version contains supplementary material available at 10.1186/s12906-024-04442-0.

## Introduction

*Melissa officinalis* L., also known as lemon balm, is a perennial medicinal plant of the Lamiaceae family that has long been used in various ethno-medicinal applications for the treatment of numerous diseases. Today, *M. officinalis* grows all over the world, while southern Europe, eastern Mediterranean region, northern Africa, western Asia and northern Iran are considered as its main regions of origin [1].

The applications of lemon balm in traditional medicine date back to more than 2000 years ago, and Theophrastus, Plinius, and Hippocrates mentioned it as a beneficial and effective herbal remedy [2]. The remarkable medicinal properties of lemon balm were first noticed when Dioscorides (1st century AD) found that a decoction of lemon balm leaves was beneficial for people bitten by scorpions, spiders, and dogs [3]. Other useful effects of lemon balm described by Dioscorides include the treatment of dysentery, suffocation caused by mushroom poisoning, difficult breathing, amenorrhea, intestinal ulcers and toothache [3]. Avicenna (Ibn Sina) has explained in his book (*Canon of Medicine*) that lemon balm can be used for phlegmatic and bilious patients suffering from depression, obsession, anxiety, and psychosis [4]. Lemon balm was also consumed in traditional medicine to treat many diseases related to the central nervous system such as migraine, sleep disorders, epilepsy, vertigo, nervousness and hysteria [5–7]. The essential oil of lemon balm and its tea are popular in Austrian folk medicine as a good herbal remedy for treating patients with digestive and nervous disorders, ulcers, arthralgia, as well as liver and biliary diseases [8–10]. In Moroccan folk medicine, lemon balm is consumed as a sedative, anti-spasmodic and cardiac tonic [11]. In addition, Avicenna recommends its consumption to clear excess black bile from the blood in the heart and to treat palpitations [12]. Therefore, its use as a cardiovascular medicine may explain the lipid-lowering effects of lemon balm.

Lemon balm leaves contain several biologically active compounds including flavonoids, monoterpenes, triterpenes, sesquiterpenes, polyphenol compounds (especially rosmarinic acid as the major chemical compound of lemon balm) and volatile compounds [13–17]. Several preclinical studies demonstrated that *M. officinalis* has a wide range of biological features including antispasmodic, antioxidant, anti-inflammatory, hypoglycemic, antidepressant, hypolipidemic, anticancer and antinociceptive activities [11, 18–26]. Several clinical studies have investigated the potential pharmaceutical properties of *M. officinalis* consumption in patients with depression and anxiety [27], type 2 diabetes [28–30], anxiety disorders and sleep disturbances [14], Alzheimer’s disease [31], borderline hyperlipidemia [32], chronic stable angina [33], arrhythmias [34], and non-alcoholic fatty liver disease [35].

Dyslipidemia is defined as an unbalanced state of lipids profile including cholesterol, TG, LDL, and HDL and is regarded as a significant independent risk factor for the incidence of atherosclerosis and subsequent cardiovascular disease (CVD). Approximately 80% of dyslipidemia prevalence is associated with unhealthy dietary behaviors and lifestyle, while the rest can be linked to family history [36, 37]. Statins are the first-line lipid-lowering medications that show several side effects including feeling abnormally tired or physical weakness, myalgia, gastrointestinal problems, sleep disorders and etc. [38–40]. . Nowadays, complementary and alternative medicine for the prevention and treatment of lipid disorders has received increasing attention. It is worth mentioning that a wealth of data from in vitro and in-vivo experiments as well as clinical studies have demonstrated the efficacy of medicinal plants and herbal medicines in regulating lipid profile [41–44]. Therefore, the objective of the present study is to perform a meta-analysis of all clinical trials that investigated the effects of *M. officinalis* on lipids profile.

## Methods

### Search strategy

The present study was designed according to the guidelines of the 2009 preferred reporting items for systematic reviews and meta-analysis (PRISMA) statement [45]. The search was conducted sequentially using electronic databases including PubMed, Scopus, and Web of Science, and the following keywords were used in the titles and abstracts of the literature to search from the beginning to October 24, 2023: (“*Melissa*” OR “Lemon balm”) AND (“hypercholesterolemia” OR “hypertriglyceridemia” OR “blood fat” OR “blood lipid” OR “lipid blood level” OR “TG” OR “triglyceride” OR “triglycerides” OR “cholesterin” OR “cholesterol” OR “cholestenone” OR “hyperlipemia” OR “high density lipoprotein cholesterol” OR “HDL"OR “low density lipoprotein cholesterol” OR “LDL”). Furthermore, the reference lists of published systematic and narrative reviews were examined to find any potential studies that were not retrieved by our electronic search. The search was restricted to English-language studies, and the search keywords are presented in Supplementary Materials Table 1.

**Table 1:** The main characteristics of the included studies

Study, Year	Study design	Follow-up	Treatment	Administered daily dose	control	Clinical outcome				Patients	No. of patients
						TC	TG	LDL	HDL		
Asadi et al. (2019)	Randomized, double-blinded, placebo-controlled study	12 weeks	Hydroalcoholic extract of *M. officinalis*	700 mg/d- two capsules per day (350 mg)	Placebo	↓	↓*	↑	↑**	Type 2 diabetes	62
Jandaghi et al. (2016)	Randomized, double-blinded, placebo-controlled study	2 months	*M. officinalis* leaf powder	3000 mg/d- three capsules per day (1000 mg)	Placebo	↓	↓	↓*	↓	Borderline hyperlipidemia	58
Javid et al. (2018)	Randomized, double-blinded, placebo-controlled study	8 weeks	*M. officinalis* leaf powder	3 g per day (3 capsules)	Placebo	↓***	↓***	↓***	↑***	Chronic stable angina	73
Kheirkhah et al. (2021)	Randomized open-label controlled trial	12 weeks	Tea Bag of *M. officinalis*	2 gr twice per day (4 gr)	Placebo	↓**	↓*	↓	↑	Premature ventricular contraction	59
Kim et al. 1800 (2023)	Randomized, double-blinded, placebo-controlled study	24 weeks	*M. officinalis* extract	1800 mg/d ALS-L1023	Placebo	↑	↓	↓	↑	Non-Alcoholic Fatty Liver Disease	33
Kim et al. 1200 (2023)	Randomized, double-blinded, placebo-controlled study	24 weeks	*M. officinalis* extract	1200 mg/d ALS-L1023	Placebo	↓*	↓*	↓*	↑	Non-Alcoholic Fatty Liver Disease	32

*Significant in *P* ≤ 0.05,

**Significant in *P* ≤ 0.01

***Significant in *P* ≤ 0.001

### Study selection

The purpose of the present metaanalysis study is to investigate the effects of *M. officinalis* (lemon balm) consumption on serum lipid profile. Clinical trials were included in the meta-analysis if they met the following inclusion criteria: [1] randomized controlled trial with either cross-over or parallel design [2], with or without blinding [3], conducted on patients with any type of disease [4], studies that provided adequate data for each group at the beginning and end of the follow-up period, or research that presented net values of change. The excluded criteria were [1] non-randomized clinical trials [2], case studies [3], uncontrolled trials [4] observational investigations with cross-sectional, case-control or cohort design [5], failure to provide necessary data at baseline or at the end of the follow-up time. The research question for the study following the population-intervention-comparator-outcome (PICO) format was: population; patients with any type of disease, intervention; *M. officinalis* (lemon balm), comparison; placebo group, and outcomes; lipid profile markers, namely high-density lipoprotein cholesterol (HDL), low-density lipoprotein cholesterol (LDL), triglyceride (TG) and total cholesterol (TC).

### Data extraction

In order to minimize bias and error in data collection, after removing duplicate studies, two researchers independently reviewed all remaining records and relevant articles were selected through primary (title and abstract) and secondary (full text) screening based on the predetermined exclusion and inclusion criteria. No studies were priorly excluded due to poor design or data quality. After evaluating all the studies, the disagreement between the evaluated results by two authors was first identified and resolved until the two reached a unanimous decision. Clinical data was robustly extracted from all eligible studies and entered into a form including first author’s name, publication year, type of study (study design), type of *M. officinalis* used in the intervention group (herbal extract, powder or etc.), administered daily dose, treatment duration, type of control group, type of disease, final clinical outcomes.

### Quality assessment

Risk of bias in the selected clinical trials for this meta-analysis was examined based on the revised Cochrane risk-of-bias tool for randomized trials (RoB 2) [46]. Bias arising from the randomization process, bias due to deviations from intended interventions, missing outcome data, measurement of the outcome, and bias in selection of the reported result for each of the included studies were assessed as low risk of bias, some concerns and high risk of bias.

### Data synthesis and primary analysis

Meta-analysis was conducted using Comprehensive Meta-Analysis (CMA) V4 software (Biostat, NJ) [47]. Results were presented in several units of measurement. For statistical analysis, the levels of the lipid profiles, comprising TC, TG, HDL, and LDL, were calculated as continuous variables and for each group, the mean or mean change was presented with its associated standard deviation (SD). The following approximations were also used: If a research provided medians and interquartile ranges, these could be converted to means and its related SD as defined by Luo et al. [48] and Wan et al. [49]. If a study provided adjusted mean in 95% confidence interval, the SD could be calculated from the confidence interval by the following equation:

$$CL = {\rm{\bar X}} \pm {\rm{Z}} \times \frac{S}{{\sqrt n }}$$

where CL, X̄, S and n represent confidence level, sample mean, standard deviation, sample size respectively. Z-value for 95% confidence interval is 1.960.

In this study, standardized mean differences (SMDs) were employed due to the various metrics that were utilized to evaluate outcomes. Therefore, the sample size, mean and standard deviation of each group (intervention and control group) for each relevant outcome were collected to calculate the SMDs. The fixed and random effects models were used based on the heterogeneity statistics including Cochran Q test and *I*_2_ index, where the data with *p*-value ≥ 0.05 and *I*_2_ ≤ 50% were specified as having low heterogeneity (fixed-effects model) and data with *p*-value < 0.05 and *I*2 > 50% were considered as having high heterogeneity (random-effects model). Possible publication bias was assessed by examining the symmetry status of Begg’s funnel plot, Begg’s rank correlation and Egger’s weighted regression tests. The sensitivity analysis was conducted using the one-study exclusion method in order to evaluate the robustness of the obtained results and verify that they were not overly affected by any particular research.

### Meta-regression

Meta-regression was performed in order to evaluate the association between calculated SMD in TG, TC, LDL and HDL values with dose, age and duration of treatment in the included studies. Meta-regression was performed under a fixed-effects or random-effects models according to the results of heterogeneity analysis and *I*_2_ values.

## Results

### Study characteristics

**Fig. 1:**
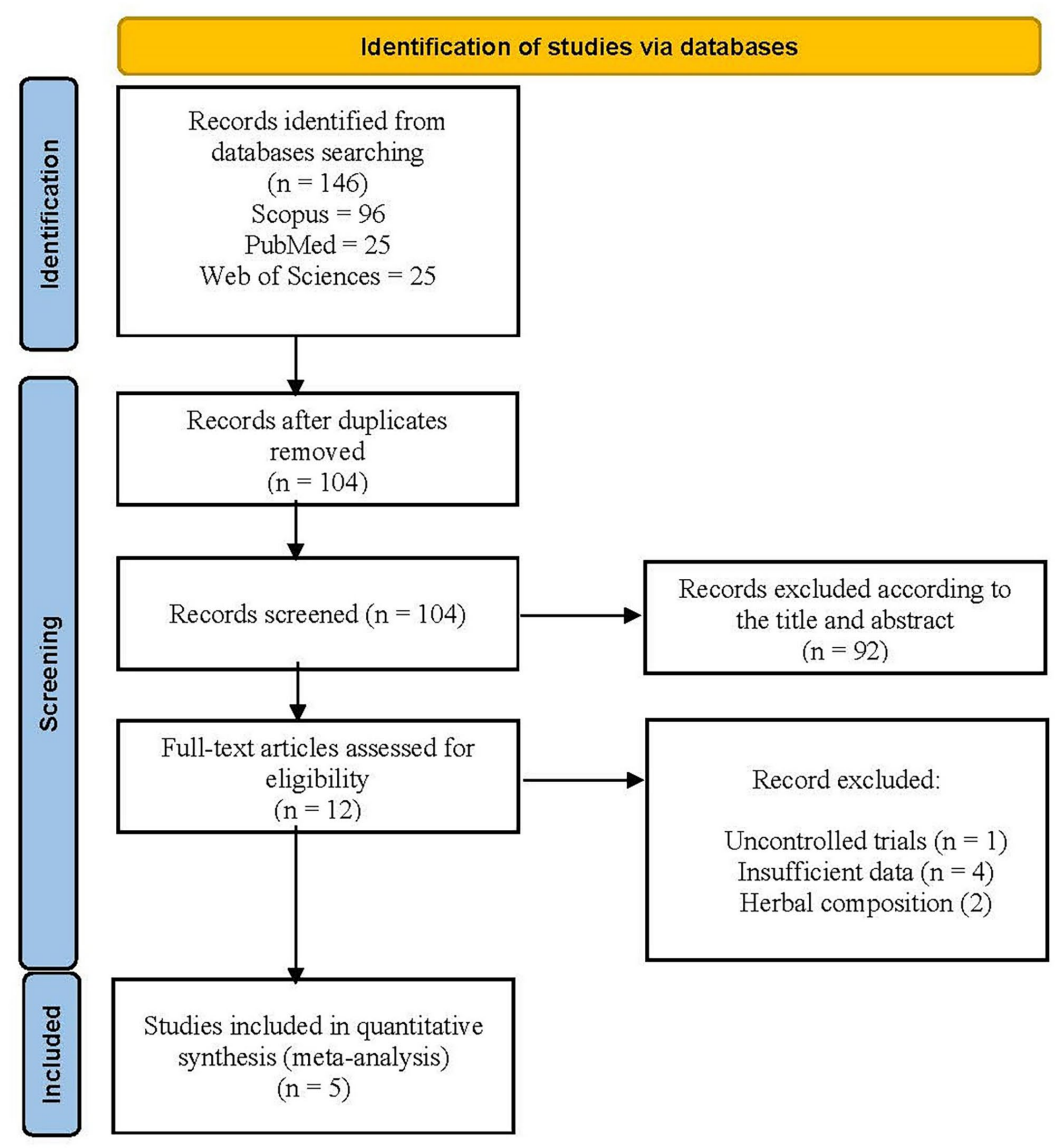
Flow diagram of study selection for meta-analysis

We identified 104 studies through systematic database search. Among the selected researches,134 studies were excluded after careful reading of the title and abstract. It was due to the presence of duplicate studies, review articles, in vitro and in vivo studies, case observations, note, letter to editor, or unrelated to the purpose of this meta-analysis study. Upon further analysis of the full text of the remaining 12 articles, 5 studies matched the inclusion criteria and were chosen for the meta-analysis. A flow chart representing our research selection procedure is depicted in Fig. 1. All 5 studies with a total of 302 patients provided data for serum TC, TG, HDL and LDL levels [30, 32–35]. The characteristics of these eligible studies are detailed in Table 1. Jandaghi et al. [32] performed randomized double-blind placebo-controlled clinical trial to evaluate the effects of *M. officinalis* leaf powder (3000 mg/d- three capsules per day (1000 mg)) on patients with borderline hyperlipidemia for 2 months. Javid et al. [33] used the same dose of *M. officinalis* leaf powder to evaluate its effects on serum biomarkers of oxidative stress, inflammation and lipid profile in patients with chronic stable angina for 8 weeks. Kheirkhah et al. used *M. officinalis* tea bag (4 gram per day) to conduct an open-label randomized clinical trial on patient with premature ventricular contraction. Asadi et al. and Kim et al. used the extract of *M. officinalis* in patients with Type 2 diabetes (for 12 weeks) and non-alcoholic fatty liver disease (for 24 weeks) respectively. Notably, Kim et al. investigated the therapeutic effects of *M. officinalis* extract at two different doses (1200 and 1800 mg/ day) on patients with non-alcoholic fatty liver disease, thus providing two series of data for meta-analysis.

### Risk of bias assessment of clinical trials

**Fig. 2:**
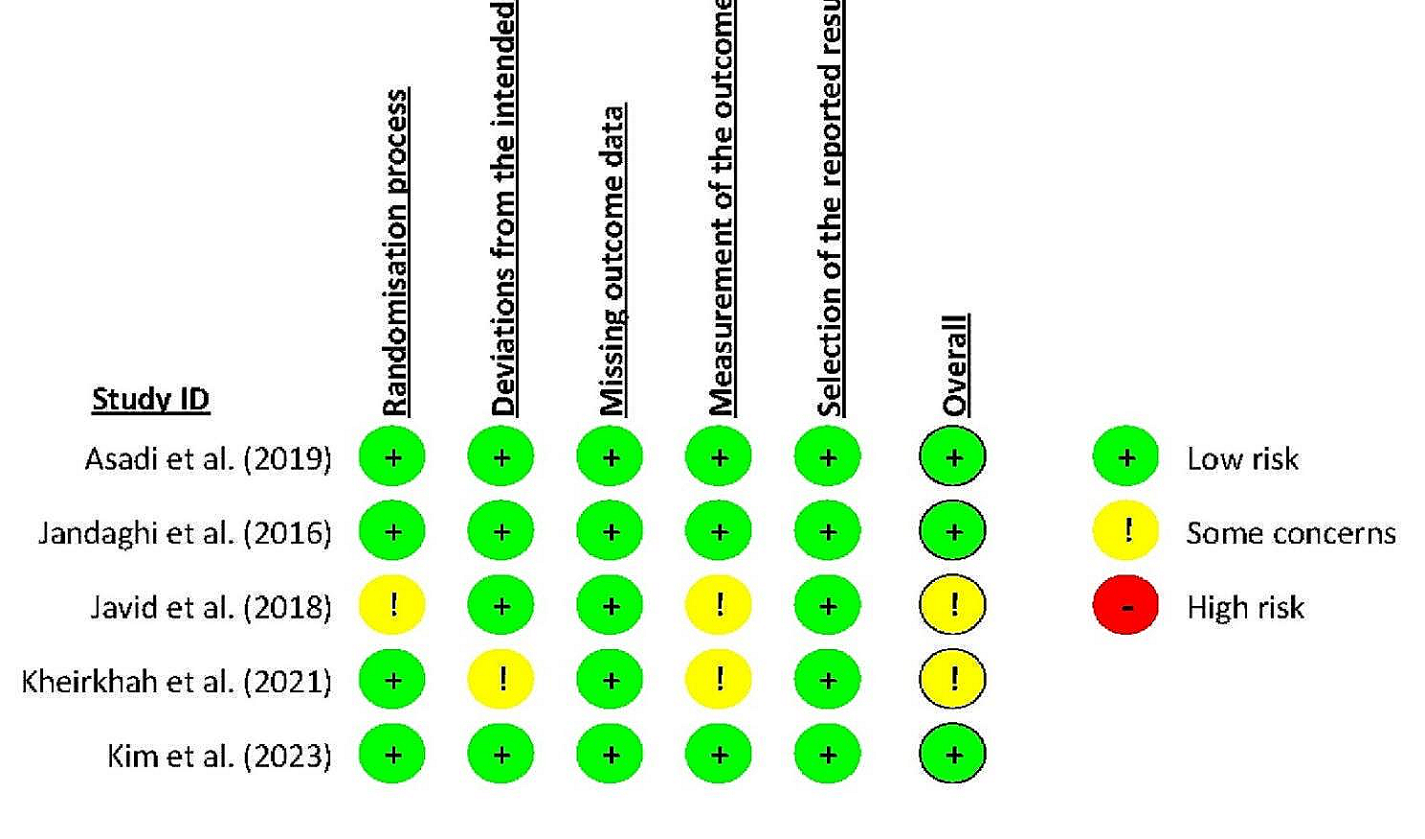
Risk of bias assessment in each study

One study of the included clinical trials revealed inadequate information regarding both random sequence generation and allocation concealment [33]. One study of the selected clinical trials lacked detailed information on randomization and allocation concealment. Two studies raised some concerns about bias arising from the outcome measurement due to lack of detailed information or open-label design of trial [33, 34]. Finally, all included clinical studies demonstrated a low risk of bias for missing outcome data and selection of the reported results. Details on the risk of bias assessment in the selected clinical studies are provided in Fig. 2.

### Effect of lemon balm on serum TG levels

**Fig. 3:**
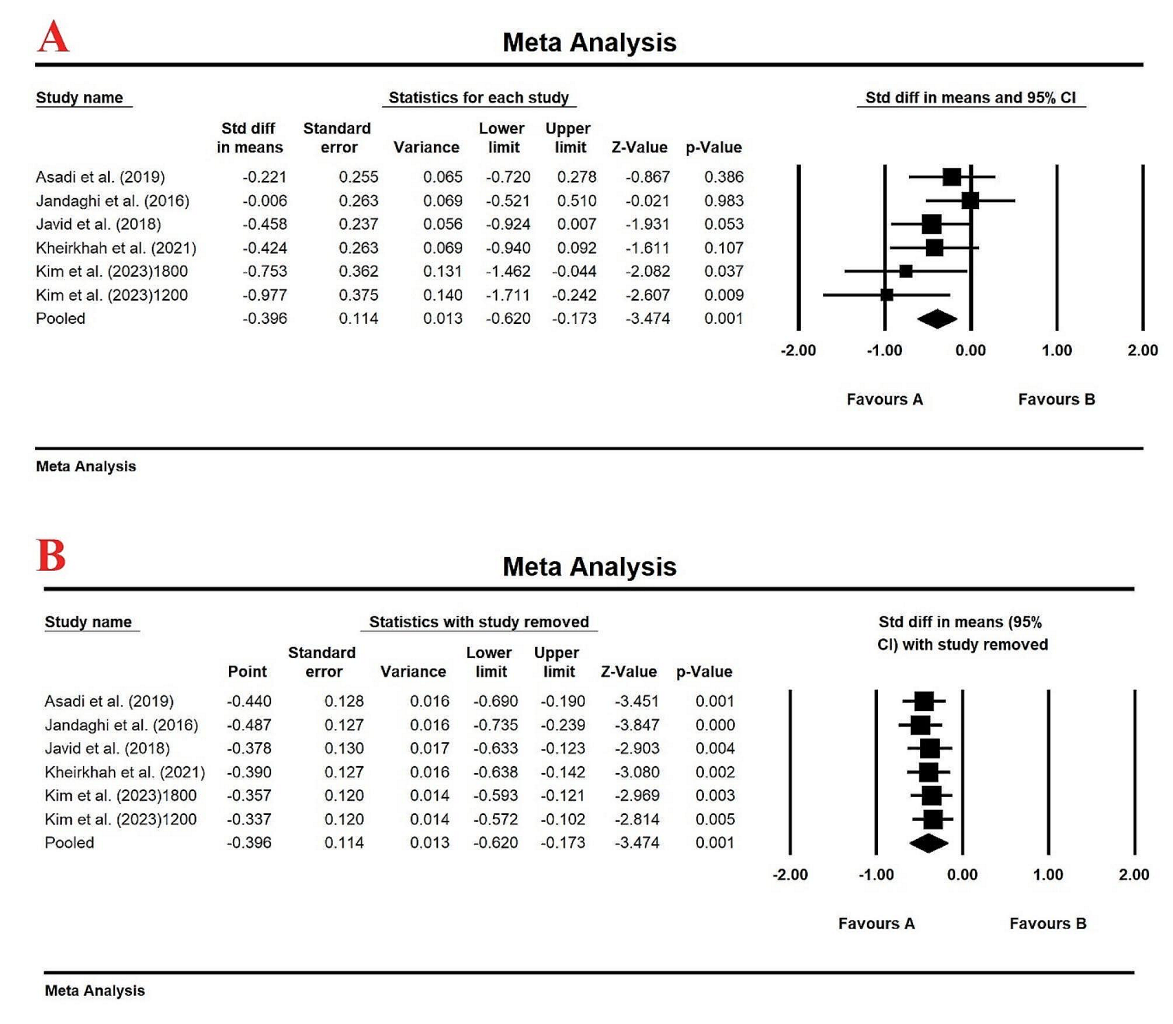
(**A**). Forest plot displaying standardized mean difference and 95% confidence intervals for the effect of lemon balm intake on TG level; (**B**). Sensitivity analysis by one-study remove method for the effect of lemon balm intake on TG level

Meta-analysis of 5 studies with a total of 302 patients demonstrated a significant decrease in serum TG levels compared to control group (SMD (95% CI): -0.396(-0.620, -0.173), *p-*value *=* 0.001) (Fig. 3A) with a Fixed-effects model (*I*_2_ = 18.53%, *p*-value = 0.293). The reduction in serum TG levels using lemon balm treatment was robust after performing sensitivity analysis by one-study remove method (Fig. 3B). Egger’s test (intercept = -4.43, standard error = 2.11; 95% CI = -10.29, 1.42, *t* = 2.10, df = 4, two-tailed *p*-value = 0.10) and upon visual inspection of the funnel plot, no evidence of publication bias was observed in clinical trials evaluating the effect of lemon balm on serum TG levels (Figure S1).

### Effect of lemon balm on serum TC levels

**Fig. 4:**
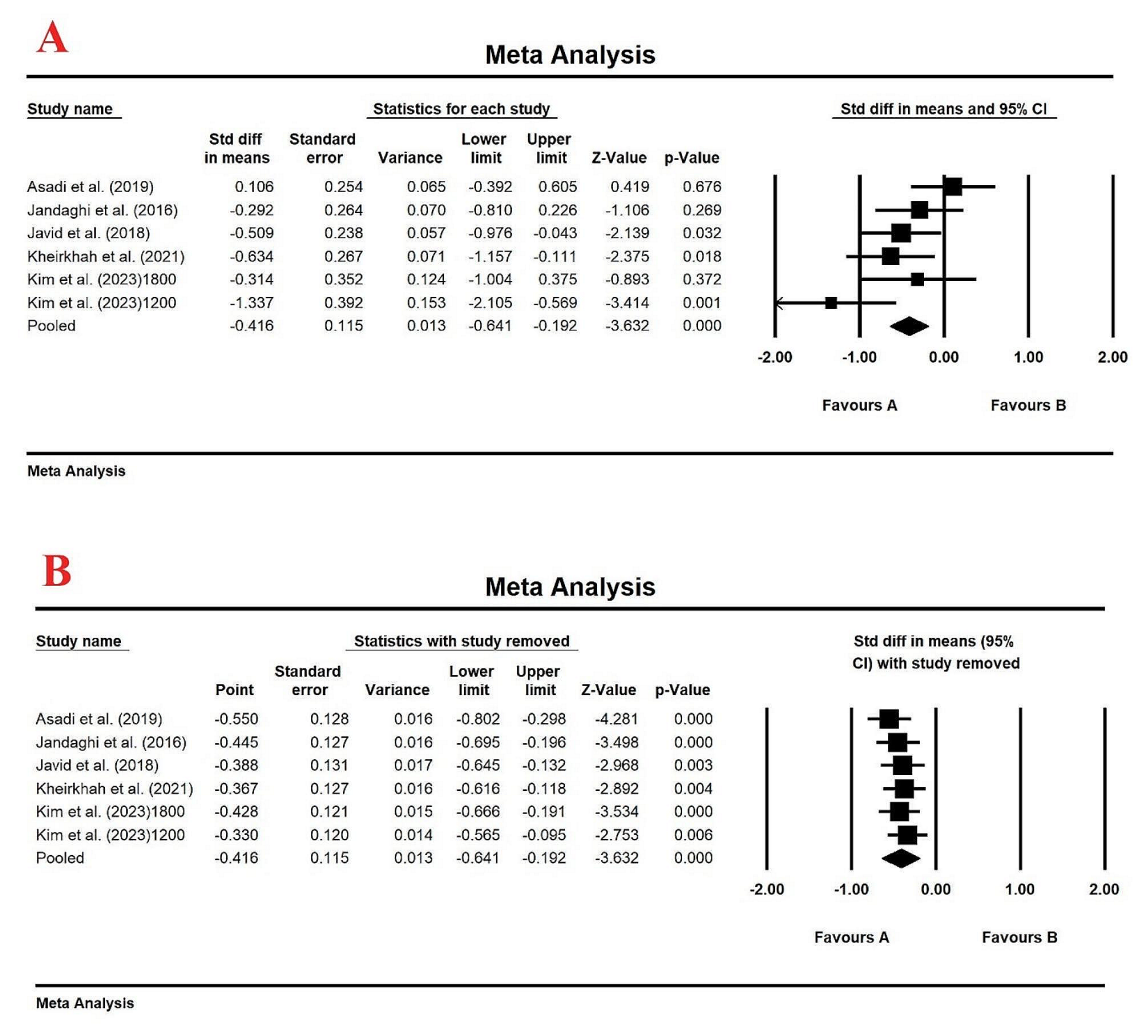
(**A**) Forest plot displaying standardized mean difference and 95% confidence intervals for the effect of lemon balm intake on TC level; (**B**). Sensitivity analysis by one-study remove method for the effect of lemon balm intake on TC level

The meta-analysis results from the 5 studies with a total of 302 patients demonstrated a significant decrease in serum TC levels compared to control group with SMD (95% CI): -0.416 (-0.641, -0.192), and *p*-value < 0.001 (Fig. 4A). The fixed-effects model was selected for this analysis (*I*_2_ = 44.03%, *p*-value 0.054). After removing any single study by sensitivity analysis, no significant changes in the overall results were observed, indicating the robustness and reliability of our findings and the fact that the data were unaffected by any particular study (Fig. 4B). Egger’s test (Intercept = -4.36, Standard error = 3.35, 95% CI = -13.68, 4.96, *t* = 1.29, df = 4.00, two-tailed *p* value = 0.26) and upon visual inspection of the funnel plot, no evidence of publication bias was observed in clinical trials evaluating the effect of lemon balm on serum TC levels (Figure S2).

### Effect of lemon balm on serum LDL levels

**Fig. 5:**
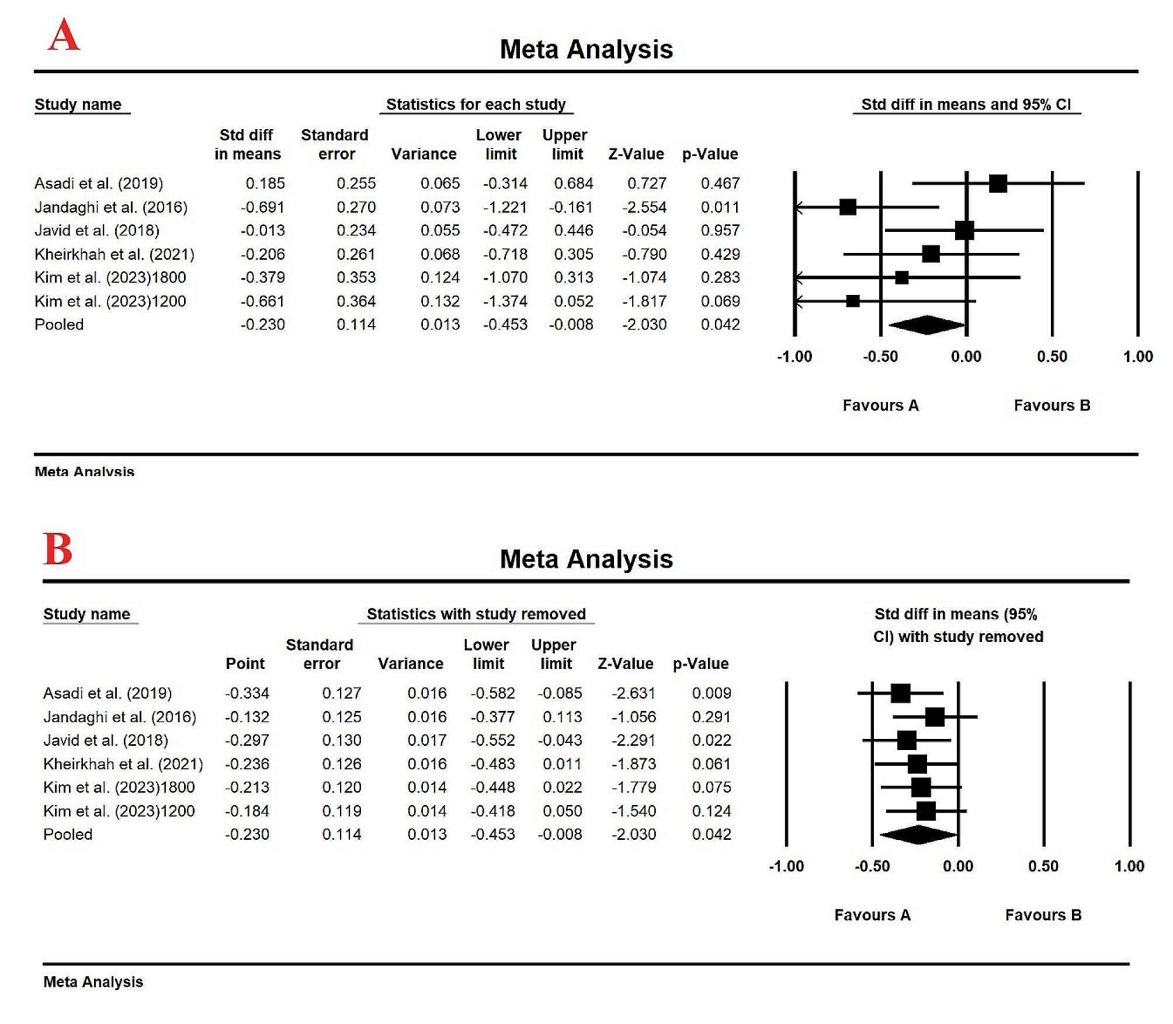
(**A**) Forest plot displaying standardized mean difference and 95% confidence intervals for the effect of lemon balm intake on LDL level; (**B**). Sensitivity analysis by one-study remove method for the effect of lemon balm intake on LDL level

The pooled results from the 5 studies with a total of 302 patients demonstrated a significant decrease in serum LDL levels compared to control group (SMD (95% CI): -0.23(-0.45, -0.008), *p* < 0.05) (Fig. 5A) with a Fixed-effects model (*I*_2_ = 37.58%, *p*-value = 0.15). After sensitivity analysis by the leave-one-out method, the reduction in serum LDL levels using lemon balm treatment was significant and the results were reliable (Fig. 5B). Egger’s test (Intercept = -4.40, Standard error = 2.95, 95% CI = -12.59, 3.79, *t* = 1.49, df = 4.00, two-tailed *p* value = 0.21) and upon visual inspection of the funnel plot, no evidence of publication bias was observed in clinical trials evaluating the effect of lemon balm on serum LDL levels (Figure S3).

### Effect of lemon balm on serum HDL levels

**Fig. 6:**
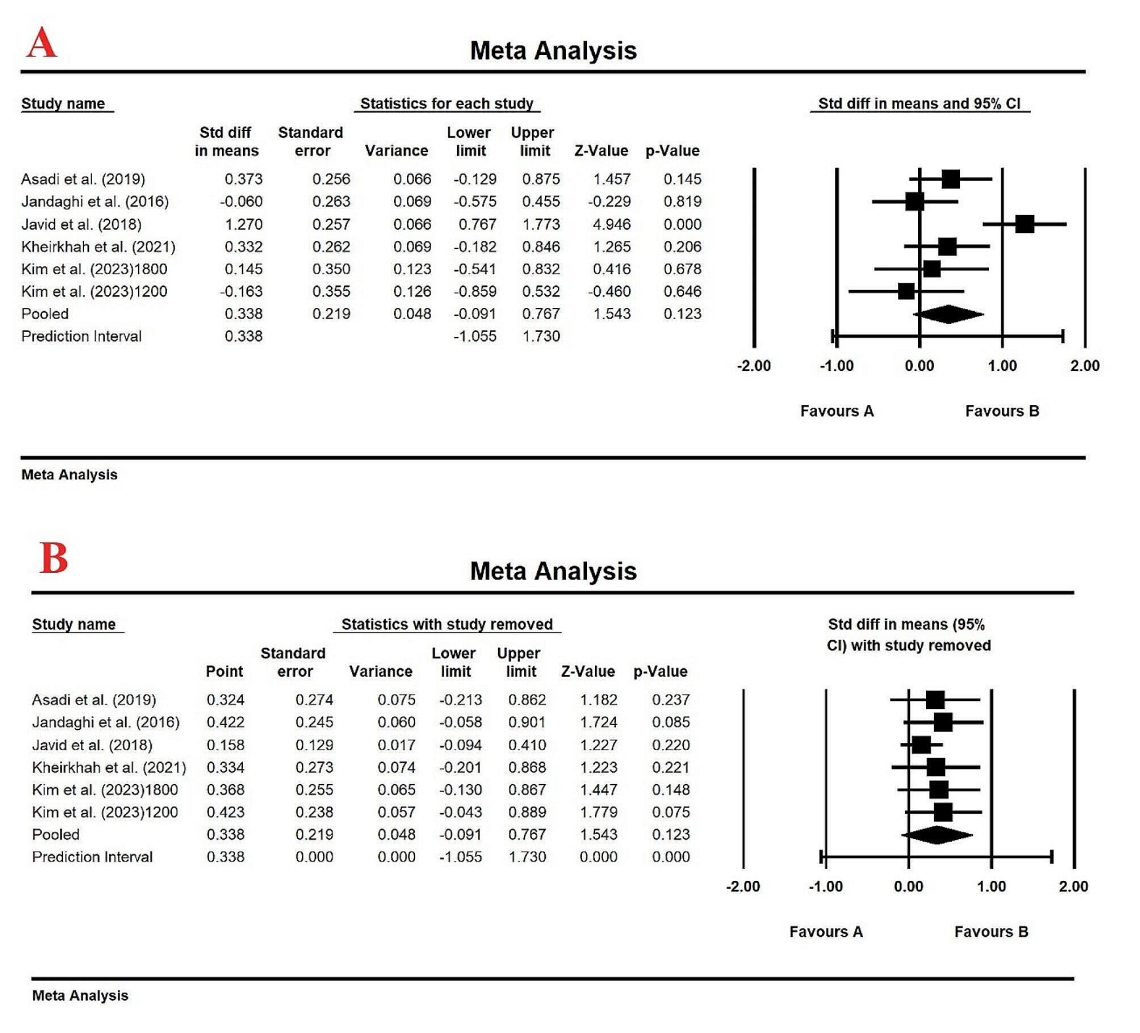
(**A**) Forest plot displaying standardized mean difference and 95% confidence intervals for the effect of lemon balm intake on HDL level; (**B**). Sensitivity analysis by one-study remove method for the effect of lemon balm intake on HDL level

The meta-analysis results from the 5 studies with a total of 302 patients did not show any considerable changes in serum HDL levels compared to control group, with SMD (95% CI): 0.336(-0.091, 0.767), prediction interval of PI (95% CI): 0.336 (-1.055, 1.730) and *p*-value of 0.123 (Fig. 6A). The random-effects model was selected for this analysis (*I*_2_ = 71.66%, *p*-value < 0.05). After removing any single study by sensitivity analysis, no significant changes in the overall results were observed, and the treatment with lemon balm had no significant effect on HDL levels (Fig. 6B). Egger’s test (Intercept = -5.86, Standard error = 5.60, 95% CI = -21.43, 9.70, *t* = 1.04, df = 4.00, two-tailed *p* value = 0.35) and upon visual inspection of the funnel plot, no evidence of publication bias was observed in clinical trials evaluating the effect of lemon balm on serum HDL levels (Figure S4).

### Meta-regression analysis

**Fig. 7:**
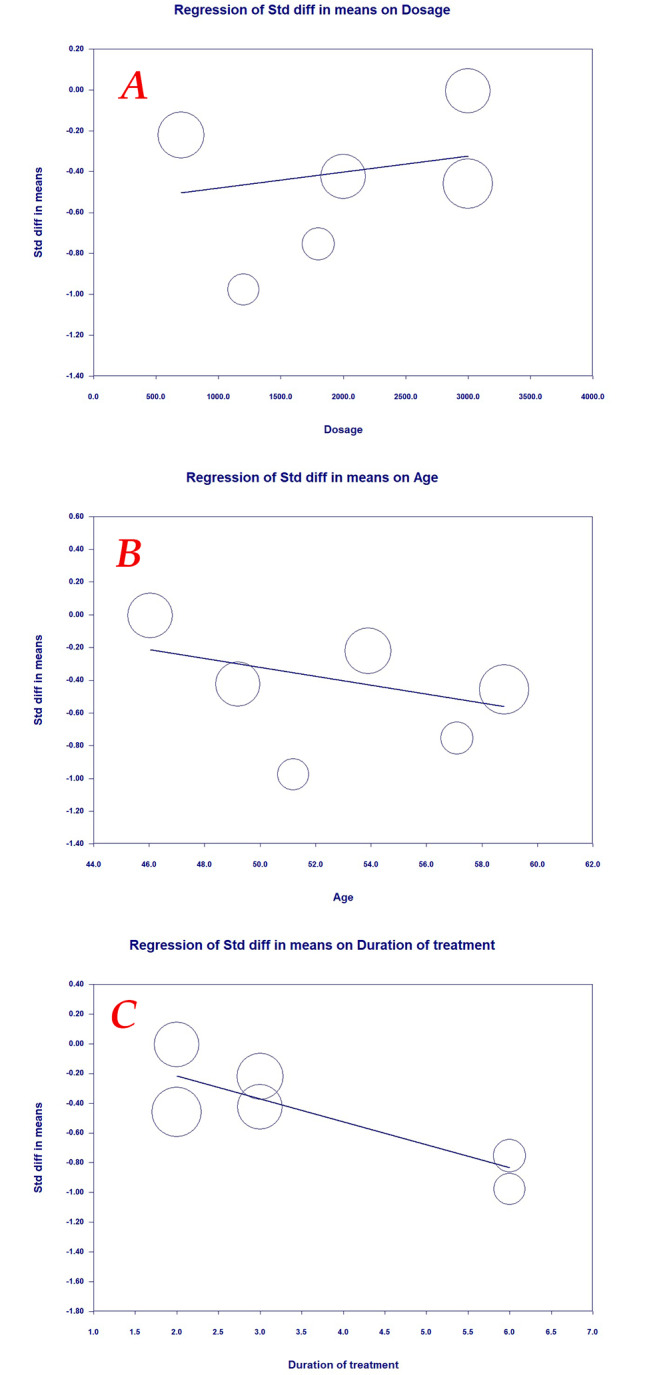
Meta-regression for assessing the effect of (**A**) Dose; (**B**) Age; (**C**) treatment duration

Potential associations between the lipid-lowering effects of lemon balm with dose, age and duration of treatment were evaluated using meta-regression analysis. The results did not suggest any significant association between lipid-lowering effects of lemon balm with dose (slope: -0.5574; 95% CI: -1.1149, 0.0000; *p* = 0.0500), age (slope: 1.0459; 95% CI: -1.4964, 3.5883; *p* = 0.4717) and duration of treatment (slope: 0.0898; 95% CI: -0.4436, 0.6232; *p* = 0.7414) (Fig. 7A, B and C).

## Discussion

Dyslipidemia can be considered as a significant preventable risk factor for atherosclerosis, CVD and type 2 diabetes. Even mild abnormalities of lipid profile may substantially increase the risk of CVD in the presence of other cardiovascular risk factors such as type 2 diabetes [50–52]. Type 2 diabetes and dyslipidemia are thought to have a reciprocal relationship, so managing one of them appropriately can have a beneficial impact on the other. Numerous studies have shown a noteworthy association between type 2 diabetes biomarkers such as HbA1c and FBS with several lipid profile parameters in diabetic patients [53–56]. Moreover, according to clinical evidence, effective management of dyslipidemia can decrease morbidity and mortality rates from coronary artery disease [57, 58]. Therefore, lowering TG, TGC, and LDL levels and raising HDL are suitable preventative measures against type 2 diabetes and cardiovascular diseases. As a result, complementary and alternative medicine has been employed extensively in research for the prevention and treatment of lipid disorders, with a growing interest being seen in the usage of medicinal plants and herbal medicines as alternative treatment worldwide.

The current meta-analysis, to our knowledge, is the first study to evaluate the effects of lemon balm treatment on serum lipid profile based on the findings of randomized clinical trials. The use of lemon balm in traditional medicine dates back to more than 20 centuries ago and is popular in folk medicine due to its various therapeutic effects. Lemon balm is listed as natural extractives generally recognized as safe (GRAS) in the USA [59]. Asadi et al. investigated the safety of lemon balm on ApoA-I, Apo B, Lipid ratio in type 2 diabetes patients. They found that taking of 700 mg/day hydroalcoholic extract of lemon balm was safe and no serious adverse effects were reported during their study [29].

The pooled analysis of data from the 5 mentioned studies, with a sample size of 302 patients showed significant decrease in TG, TC and LDL levels. While the results of lemon balm treatment on HDL reduction were not statistically significant. The estimated effect sizes for lemon balm treatment on lipid profile changes were not sensitive to any specific study. In addition, the heterogeneity of studies was assessed by the *I*^*2*^ index values and Cochran Q test, which for TG, TC and LDL the *I*^*2*^ index value was ranged between 18 and 44%. The data with *p*-value ≥ 0.05 and *I*^2^ ≤ 50% were specified as having low heterogeneity and fixed-effects model was used for meta-analysis. Also, the fixed-effect model assumes one true effect size underlies all the studies in the meta-analysis. However, after removing Kim et al. study (both arms), the result was still significant for TG and TC levels and consistent with the results of the sensitivity analysis, while the results of lemon balm treatment on LDL reduction were not statistically significant.

According to the finding of preclinical studies, lemon balm essential oil and extracts showed a significant increase in the expression levels of peroxisome proliferator-activated receptors including PPARα and PPARγ which play an important role in regulating lipid and glucose metabolism. Furthermore, the expression levels of sterol regulatory element-binding protein (SREBP)-1c and 3-hydroxy-3-methyl-glutaryl (HMG)-CoA reductase which participates in regulating the fatty acid biosynthesis process were affected by lemon balm essential oil and extracts [26, 60, 61].

The present meta-analysis is the first research that summarizes the findings of previous studies on effects of lemon balm treatment on lipid profiles. Hence, it is necessary to consider some limitations of this meta-analysis while interpreting results and drawing conclusions. The main concern of our research is the limited number of eligible studies and the relatively small population size of each individual study. The patients of these studies had different types of diseases and metabolic syndromes. However, the meta-analysis was sufficiently powered to detect the considerable effects of lemon balm in the combined population regardless of type of diseases. Another limitation of this meta-analysis was the different metrics used to evaluate outcomes, so the use of SMD as a summary statistic for the combined effect size in this meta-analysis is justified. Studies with different sample sizes and populations were included and language restrictions were considered in our study selection. Furthermore, due to heterogeneity among studies, which is evident from variations in the length of treatments, dosages, and frequency of lemon balm administration, results should be interpreted with caution. Despite these limitations, our meta-analysis provides noteworthy insights into the effects of lemon balm on serum lipid profile, but interpreting the results with caution is necessary given the acknowledged limitations. The purpose of meta-analysis studies is to systematically assess the results of previous research to derive conclusions about that body of research. Outcomes from a meta-analysis may include a more precise estimate of the effect of treatment or risk factor for disease, or other outcomes, than any individual study contributing to the pooled analysis. However, the results of this meta-analysis should be interpreted with caution for the effects of lemon balm on HDL and LDL levels. In addition, investigating the mechanism of the effect of this medicinal plant on the lipid profile requires further studies.

## Conclusion

The findings of this meta-analysis provide evidence that lemon balm may be administered as a safe and beneficial herbal medicine for reducing TC, TG and LDL levels. According to the pooled results of 5 studies with a total of 302 patients, lemon balm intake had no significant effect on HDL level. This study offers key information about the substantial impact of lemon balm on serum lipid profile, as a potential remedy in cases of dyslipidemia. It is imperative to conduct further research in order to identify the possible mechanisms behind these beneficial results.

### Electronic supplementary material

Below is the link to the electronic supplementary material.

Supplementary Material 1

## Data Availability

The datasets used and/or analyzed during the current study available from the corresponding author on reasonable request.

## Abbreviations

CMA: Comprehensive Meta-Analysis
CVD: Cardiovascular Disease
HDL: High-Density Lipoprotein Cholesterol
HMG-CoA: 3-Hydroxy-3-Methylglutaryl Coenzyme A
LDL: Low-Density Lipoprotein Cholesterol
PPAR: Peroxisome Proliferator-Activated Receptors
SD: Standard Deviation
SMDs: Standardized Mean Differences
SREBP-1c: Sterol Regulatory Element-Binding Protein
TC: Total Cholesterol
TG: Triglyceride
