# Supplementary material for: Effects of Melissa officinalis (lemon balm) consumption on serum lipid profile: a meta-analysis of randomized controlled trials
Source: BMC Complement Med Ther. 2024 Apr 4;24:146. doi: 10.1186/s12906-024-04442-0 (PMC10996117; doi:10.1186/s12906-024-04442-0)
Supplement: Supplementary file 1 — Supplementary Material 1 [file 12906_2024_4442_MOESM1_ESM.docx]

**Effects of** ***Melissa officinalis* (lemon balm) consumption on serum lipid profile: A systematic review and meta-analysis of randomized controlled trials**

Kasra Shahsavari^1^, Mohammad Reza Shams Ardekani^2^, Mahnaz Khanavi^2^, Tannaz Jamialahmadi^3,4^, Mehrdad Iranshahi^3^, Maede Hasanpour^2,3^^[[1]](#footnote-1)^*

*^1^School of Medicine, Tehran University of Medical Sciences, Tehran, Iran*

*^2^Department of Pharmacognosy, Faculty of Pharmacy, and Persian Medicine and Pharmacy Research Center, Tehran University of Medical Sciences, Tehran, Iran.*

*^3^Biotechnology Research Center, Pharmaceutical Technology Institute, Mashhad University of Medical Sciences, Mashhad, Iran*

*^4^Department of Nutrition, Faculty of Medicine, Mashhad University of Medical Sciences, Mashhad, Iran*


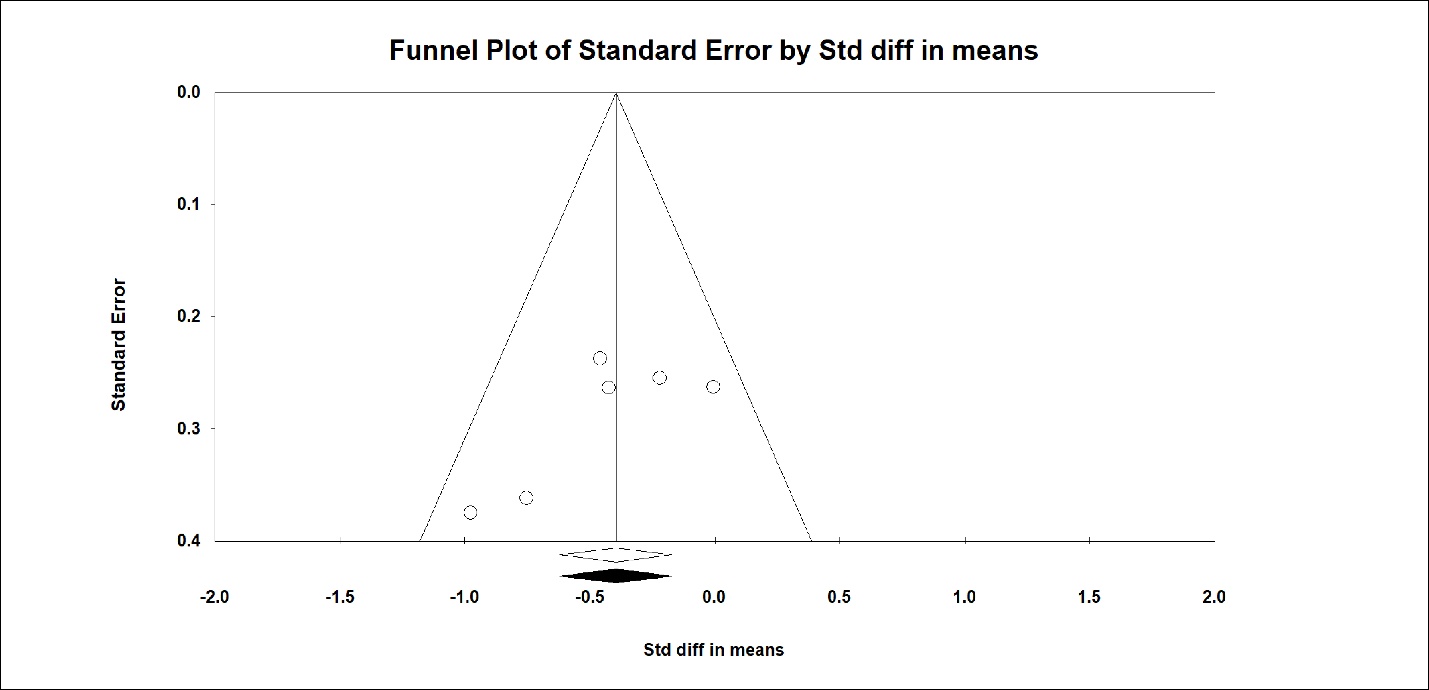


**Figure S1:** Publication bias funnel plots of studies that reported the effect of lemon balm treatment on TG level.


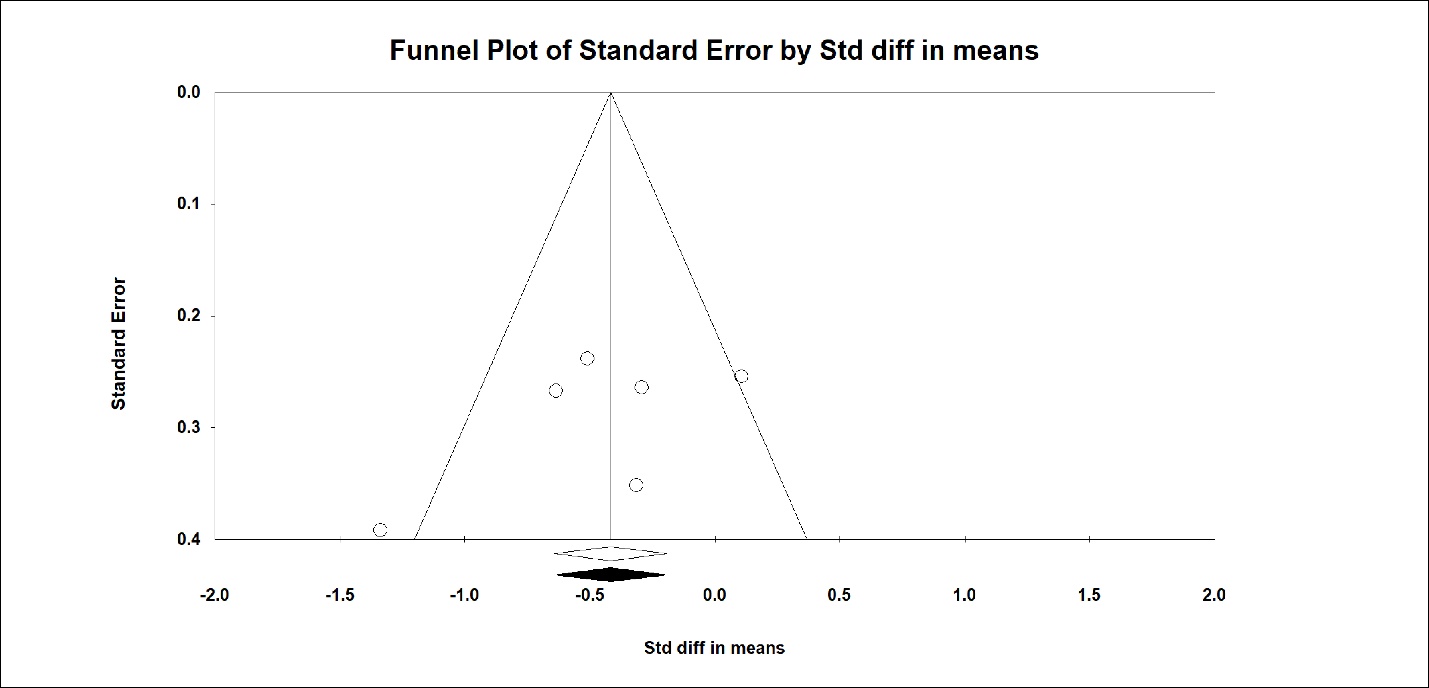


**Figure S2:** Publication bias funnel plots of studies that reported the effect of lemon balm treatment on TC level


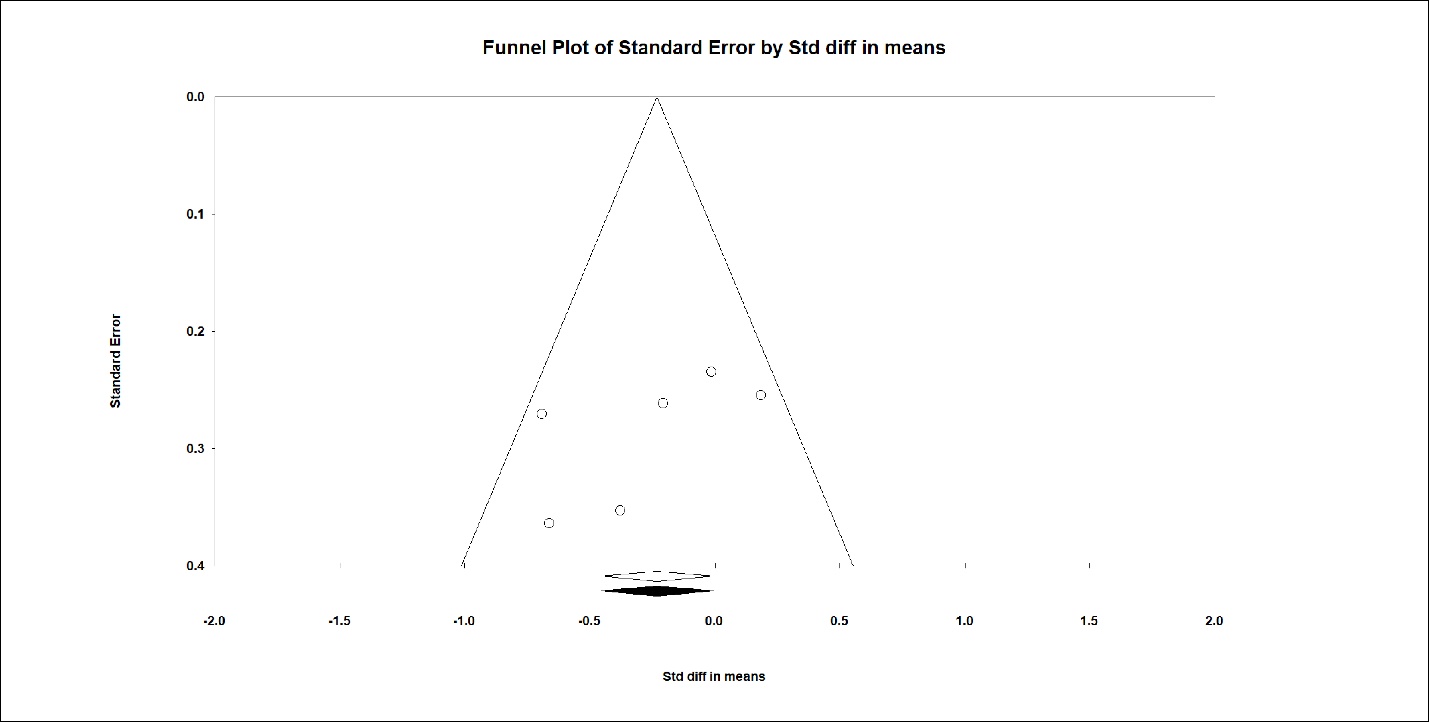


**Figure S3:** Publication bias funnel plots of studies that reported the effect of lemon balm treatment on LDL level


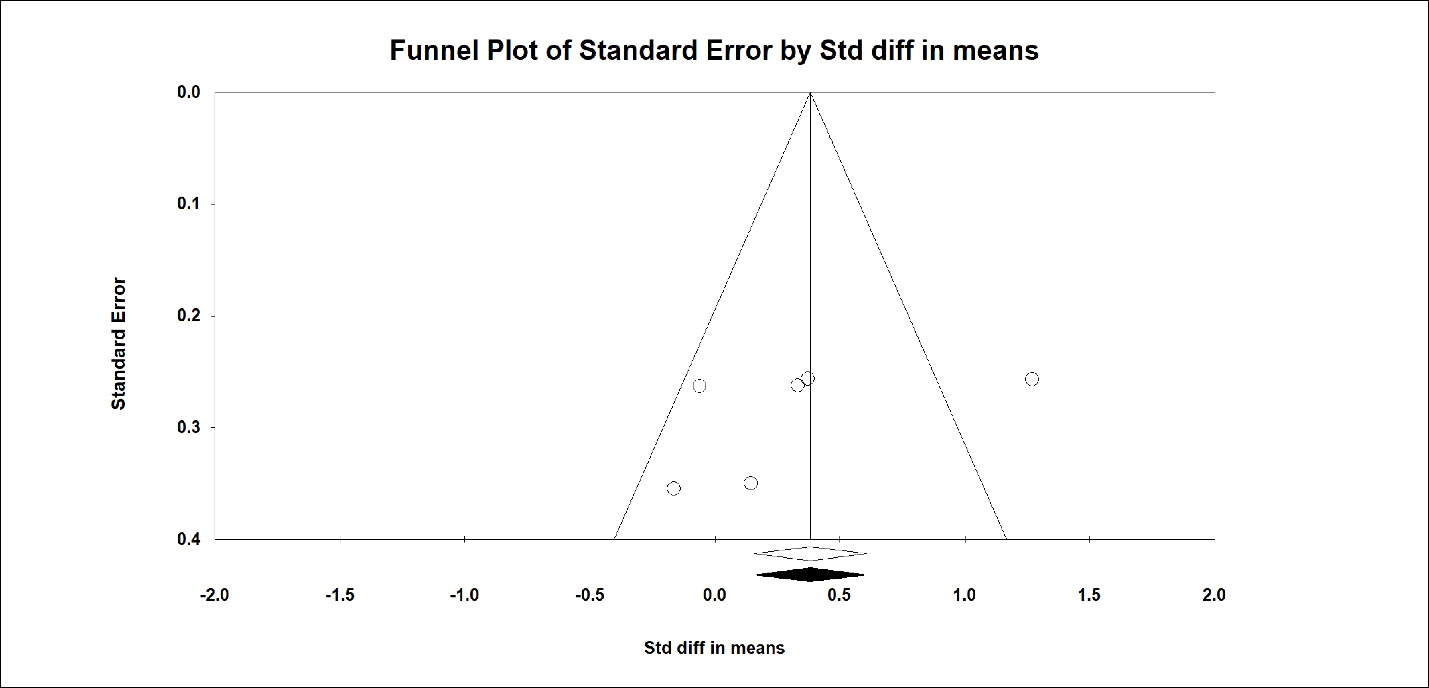


**Figure S4:** Publication bias funnel plots of studies that reported the effect of lemon balm treatment on HDL level

**Table S1:** The search terms of this study

| Databases | Keywords | No |
| --- | --- | --- |
| Scopus | ( TITLE-ABS-KEY ( melissa ) OR TITLE-ABS-KEY ( lemon AND balm ) AND TITLE-ABS-KEY ( hypercholesterolemia ) OR TITLE-ABS-KEY ( hypertriglyceridemia ) OR TITLE-ABS-KEY ( blood AND fat ) OR TITLE-ABS-KEY ( blood AND lipid ) OR TITLE-ABS-KEY ( lipid AND blood AND level ) OR TITLE-ABS-KEY ( TG ) OR TITLE-ABS-KEY ( triglyceride ) OR TITLE-ABS-KEY ( triglycerides ) OR TITLE-ABS-KEY ( glycerin AND trilaurate ) OR TITLE-ABS-KEY ( cholesterin ) OR TITLE-ABS-KEY ( cholesterol ) OR TITLE-ABS-KEY ( cholestenone ) OR TITLE-ABS-KEY ( hyperlipemia ) OR TITLE-ABS-KEY ( high AND density AND lipoprotein AND cholesterol ) OR TITLE-ABS-KEY ( HDL ) OR TITLE-ABS-KEY ( low AND density AND lipoprotein AND cholesterol ) OR TITLE-ABS-KEY ( LDL ) ) | 96 |
| PubMed | ("Melissa"[Title/Abstract] OR "Lemon balm"[Title/Abstract]) AND ("hypercholesterolemia"[Title/Abstract] OR "hypertriglyceridemia"[Title/Abstract] OR "blood fat"[Title/Abstract] OR "blood lipid"[Title/Abstract] OR "lipid blood level" [Title/Abstract] OR "TG"[Title/Abstract] OR "triglyceride"[Title/Abstract] OR "triglycerides" [Title/Abstract] OR "cholesterin"[Title/Abstract] OR "cholesterol"[Title/Abstract] OR "cholestenone"[Title/Abstract] OR "hyperlipemia"[Title/Abstract] OR "high density lipoprotein cholesterol"[Title/Abstract] OR "HDL"[Title/Abstract] OR "low density lipoprotein cholesterol"[Title/Abstract] OR "LDL"[Title/Abstract]) | 25 |
| Web of Sciences | TS=((("Melissa") OR ("Melon balm")) AND (("hypercholesterolemia") OR ("hypertriglyceridemia") OR ("blood fat") OR ("blood lipid") OR ("lipid blood level") OR ("TG") OR ("triglyceride") OR ("triglycerides") OR ("cholesterin") OR ("cholesterol") OR ("cholestenone") OR ("hyperlipemia") OR ("high density lipoprotein cholesterol") OR ("HDL") OR ("low density lipoprotein cholesterol") OR ("LDL"))) | 25 |

1. * **Corresponding Author:**

   **Maede Hasanpour**

   Department of Pharmacognosy, Faculty of Pharmacy, and Persian Medicine and Pharmacy Research Center, Tehran University of Medical Sciences, Tehran, Iran.

   Biotechnology Research Center, Pharmaceutical Technology Institute, Mashhad University of Medical Sciences, Mashhad, Iran

   Phone: +98-21-64122448, Fax: +98-21-64120000

   E-mail: [mhasanpour@farabi.tums.ac.ir](mailto:mhasanpour@farabi.tums.ac.ir) and [maede.hasanpour@yahoo.com](mailto:maede.hasanpour@yahoo.com) [↑](#footnote-ref-1)
